# The New Poor

**Published:** 1920-05-15

**Authors:** 


### THE NEW POOR.

### Hospital and Nursing Home Accommodation.

On several occasions the question of hospital and nursing home accommodation has been discussed in these columns from the standpoint of patients with moderate incomes. Their case is undoubtedly hard, and the problem arises in every part of the country. The *Glasgow Herald* has given valuable attention to the subject, and has published useful information about attempts made in some of the great cities to meet the difficulty.

In London two of the largest hospitals, Guy's and St. Thomas's, have laid themselves out to take in patients paying moderate fees. At Guy's one large hospital ward has been allocated for this purpose and divided up into twenty-four cubicles—fourteen for men and ten for women—with one private room. At St. Thomas's a special home has been built in the hospital grounds. There are forty beds, screened off from each other by curtains. Guy's scheme has been in operation since 1912, and each year has afforded a fairly good surplus of receipts over expenditure. At St. Thomas's also

THE PUBLIC WELL-BEING—(*continued*).

the expenditure has been met by the receipts, and any surplus has been handed over to the general hospital funds. The schemes adopted in these institutions appear to have proved advantageous both to the hospitals themselves as well as to the class of patients for whom the accommodation was specially provided. So numerous are the applications for admission that "beds are booked up long ahead." Another hospital, Mildmay, is to be rebuilt, and the Council is appealing for £50,000 for the purpose. The object in view in rebuilding the hospital is to provide 110 beds for the "new poor"—middle-class patients of good birth and education whose strictly limited incomes are totally inadequate to meet the expenses of a nursing home. It is estimated that if the patients pay an average fee of 2½ guineas per week the hospital eventually will be self-supporting.

In Liverpool the principal hospitals all have a few private wards in which the patients pay a minimum fee of £3 3s. a week, the surgeon or physician making his own arrangements for his fee.

In Manchester one of the smaller hospitals has already opened its wards to paying patients, and at the principal hospital, the Royal Infirmary, a small committee is at present sitting to consider the question of the admission of patients of limited means. In addition, however, to hospital accommodation, there is a private nursing home, containing nineteen beds and a cot in wards, founded for the express purpose of treating patients of the class under discussion. So successful has this home proved and so great is the pressure of applications for admission that the governing body are contemplating enlarging the present premises or removing to more commodious ones.

In Birmingham a special hospital, St. Chad's, containing 100 beds, has been constructed. The patients pay an "inclusive" fee, a fee which covers all residential and professional expenses, the amount being determined by the circumstances of the case. Five years' practical experience of the scheme has shown it to be abundantly successful.

Edinburgh, like Manchester, has launched forth into the institution of a special home exclusively devoted to the purpose of admitting patients only capable of paying considerably modified fees. It has done so, also, with an equal amount of success. This particular home affords accommodation for about fifty patients, possessing as many as fourteen single rooms. The charges run from £2 to £4 10s. The home is in every way admirably equipped, and approaches as near as possible to meeting the varied surgical and medical requirements only to be found efficiently supplied in our large public hospitals. So well patronised is this home both by the profession and patients—for there is a "waiting list"—that it is enabled annually to very materially lessen the considerable expenditure involved in the original outlay for the extensive alterations made to meet the requirements aimed at.

The writer of the article which gives these and other details comes to the conclusion that projects of this kind must be self-supporting once the initial outlay has been made, and the examples given show credit balances in every case. Two possible ways of meeting the present requirements are thus to provide accommodation in existing hospitals (or to erect other buildings), and to provide special homes. The one is not antagonistic to the other, and neither is antagonistic to public hospitals and private nursing homes. Patients are not admitted unless they are entitled to admission on financial grounds. So far as the general practitioner is concerned, the homes are better, because he can there bring in his own patient and manage the case himself.

